# Swarming Magnetic Fe_3_O_4_@Polydopamine-Tannic Acid Nanorobots: Integrating Antibiotic-Free Superficial Photothermal and Deep Chemical Strategies for Targeted Bacterial Elimination

**DOI:** 10.34133/research.0438

**Published:** 2024-07-31

**Authors:** Luying Si, Shuming Zhang, Huiru Guo, Wei Luo, Yuqin Feng, Xinkang Du, Fangzhi Mou, Huiru Ma, Jianguo Guan

**Affiliations:** ^1^ State Key Laboratory of Advanced Technology for Materials Synthesis and Processing, International School of Materials Science and Engineering, Wuhan University of Technology, Wuhan, China.; ^2^ Wuhan Institute of Photochemistry and Technology, Wuhan, China.; ^3^ School of Chemistry, Chemical Engineering and Life Science, Wuhan University of Technology, Wuhan, China.

## Abstract

Micro/nanorobots (MNRs) are envisioned to provide revolutionary changes to therapies for infectious diseases as they can deliver various antibacterial agents or energies to many hard-to-reach infection sites. However, existing MNRs face substantial challenges in addressing complex infections that progress from superficial to deep tissues. Here, we develop swarming magnetic Fe_3_O_4_@polydopamine-tannic acid nanorobots (Fe_3_O_4_@PDA-TA NRs) capable of performing targeted bacteria elimination in complicated bacterial infections by integrating superficial photothermal and deep chemical strategies. The Fe_3_O_4_@PDA-TA nanoparticles (NPs), serving as building blocks of the nanorobots, are fabricated by in situ polymerization of dopamine followed by TA adhesion. When driven by alternating magnetic fields, Fe_3_O_4_@PDA-TA NPs can assemble into large energetic microswarms continuously flowing forward with tunable velocity. Thus, the swarming Fe_3_O_4_@PDA-TA NRs can be navigated to achieve rapid broad coverage of a targeted superficial area from a distance and rapidly eradicate bacteria residing there upon exposure to near-infrared (NIR) light due to their efficient photothermal conversion. Additionally, they can concentrate at deep infection sites by traversing through confined, narrow, and tortuous passages, exerting sustained antibacterial action through their surface TA-induced easy cell adhesion and subsequent membrane destruction. Therefore, the swarming Fe_3_O_4_@PDA-TA NRs show great potential for addressing complex superficial-to-deep infections. This study may inspire the development of future therapeutic microsystems for various diseases with multifunction synergies, task flexibility, and high efficiency.

## Introduction

Bacterial infections are one of the main causes of worldwide morbidity and death [1]. Using antibiotics is considered an efficient sterilization technology, but excessive use will increase antimicrobial resistance (AMR) and threaten human health [2]. Therefore, it is of great significance to develop non-antibiotic antimicrobial strategies [3,4]. As alternatives to antibiotics, antimicrobial peptides (AMPs) exhibiting membrane disruption and immunomodulatory ability have been developed as a common and effective strategy [5]. In addition, engineered phage, specific vaccines and quorum sensing (QS) inhibitors have been exploited to assist antibiotic therapy [6]. Unsatisfactorily, these biological agents are limited by high cost and usually show their effect only on specific infection strains. Furthermore, chemical dynamic therapy (CDT) [7], photothermal therapy (PTT) [8], photodynamic therapy (PDT) [9], and sonodynamic therapy (SDT) [10] are novel therapeutic strategies in fighting against multidrug-resistant (MDR) bacteria. As generally acknowledged, PTT is a promising physical therapy with low invasion and independence on the local concentration of chemicals in infection microenvironments. Among various photothermal agent (PTA), polydopamine (PDA) has been used as a biocompatible platform benefiting from its broad-band light absorption properties and great flexibility to be modified and incorporated with other antibacterial and anticancer agents [11–14]. Additionally, natural antibacterial-active agents including polysaccharides (e.g., chitosan) and small antibacterial molecules (e.g., polyphenols) show great potential in trauma-related infections [15,16]. Among them, tannic acid (TA) shows inherent broad-spectrum antibacterial activity and has been widely used in antibacterial materials by incorporating with hydrogels [17,18] or serving as chelating agents in metal polyphenol networks (MPNs) [19,20]. However, PTT efficacy is often limited in superficial tissue because of the limited penetration depth of light in tissues (e.g., ~1 cm for 808-nm near-infrared [NIR] light [21]). Natural antibacterial polyphenols as chemo-pharmaceuticals, on the other hand, hold promise for deep tissue treatment, but effectively delivering these molecules with high targeting efficiency remains a significant challenge.

Micro/nanorobots (MNRs) are micro/nanoscale machines capable of propelling in liquid media by harvesting energies from surrounding chemical fuels or external fields [22,23]. Benefitting from their small sizes and motility, they are expected to navigate in many hard-to-reach narrow biological environments to perform designated biomedical tasks, and thus may provide revolutionary changes to microsurgery, disease diagnosis, medical imaging, and targeted therapy [24–26]. As the collective of single MNRs, the MNR swarms show emerging collective behaviors that individual robots lack, such as powerful thrust, high robustness, dynamic reconfigurations, high imaging contrast, and collective intelligence [27–32]. Especially, the swarming magnetic nanorobots exhibit high biocompatibility and excellent adaptability to biological environments due to their fuel-free propulsion and high ion tolerance [33–40]. Thus, they show great promise in targeted eliminations of bacteria in medical catheters, root canals, biliary stents, tympanostomy tubes, and superficial wounds through mechanical destruction, magnetothermal effects, antibiotics delivery, catalytic generation of reactive oxygen species (ROS), and synergistic combinations thereof [41–47]. Nevertheless, these magnetic nanorobots are mainly designed to eliminate bacteria or biofilms on the surface of medical devices and implants or in superficial wounds, and may not be applicable in complicated bacterial infection scenarios involving both superficial and deep-seated tissues.

**Fig. 1.**
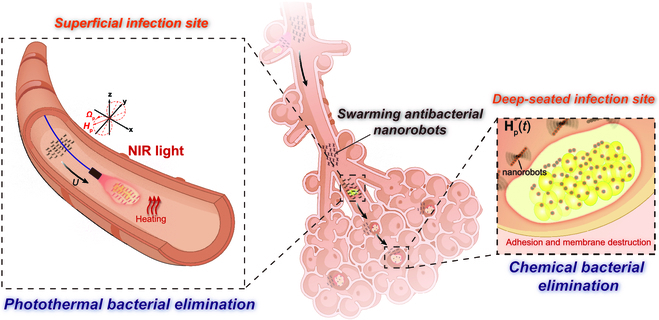
Schematic illustration of swarming Fe_3_O_4_@PDA-TA NRs performing superficial photothermal (left inset) and deep chemical (right inset) bacterial eliminations. The lung infection lesion extending from the airways to the alveoli is depicted as an example of a typical potential application scenario.

Here, we propose a simple design of swarming magnetic nanorobots integrating antibiotic-free superficial photothermal and deep chemical antibacterial activities (Fig. 1). The building blocks of the swarming magnetic nanorobots are designed as Fe_3_O_4_ nanoparticles coated with PTA PDA and natural antibacterial agent TA (Fe_3_O_4_@PDA-TA NPs). When actuated by an alternating magnetic field, such as rotating (**H**_r_(*t*)) or precessing magnetic field (**H**_p_(*t*)), Fe_3_O_4_@PDA-TA NPs self-assemble into rolling or wobbling nanochains (i.e., nanorobots) that translate in close proximity to the substrate utilizing local shear gradients [48]. Under local hydrodynamic interactions, these Fe_3_O_4_@PDA-TA NRs further organize into large microswarms while rotating around their center of mass [49]. With the powerful collective motion, the microswarm can continuously flow forward to cover a targeted area with bacterial infections from a distance and even penetrate and enter confined, narrow infection sites within deep tissues. Subsequently, they can perform superficial photothermal and deep chemical bacterial elimination simultaneously or sequentially. Specifically, Fe_3_O_4_@PDA-TA NRs covering the target area, upon exposure to NIR light, can be triggered to perform localized photothermal ablation, rapidly eradicating bacteria residing on the surface of the infection site. Meanwhile, within deep-seated infection sites, despite the hindered photothermal efficacy due to limited penetration or significant attenuation of NIR light, Fe_3_O_4_@PDA-TA NRs can exert sustained antibacterial action through their inherent chemical activities. This means that Fe_3_O_4_@PDA-TA NRs, with integrated swarming motions, photothermal conversion, and chemical antibacterial effect, show great potential to treat complicated infection lesions involving both superficial and deep-seated infection sites, such as surgical site, trauma wound, deep burn, and lung infections.

## Results

### Fabrication and characterization of building blocks

**Fig. 2.**
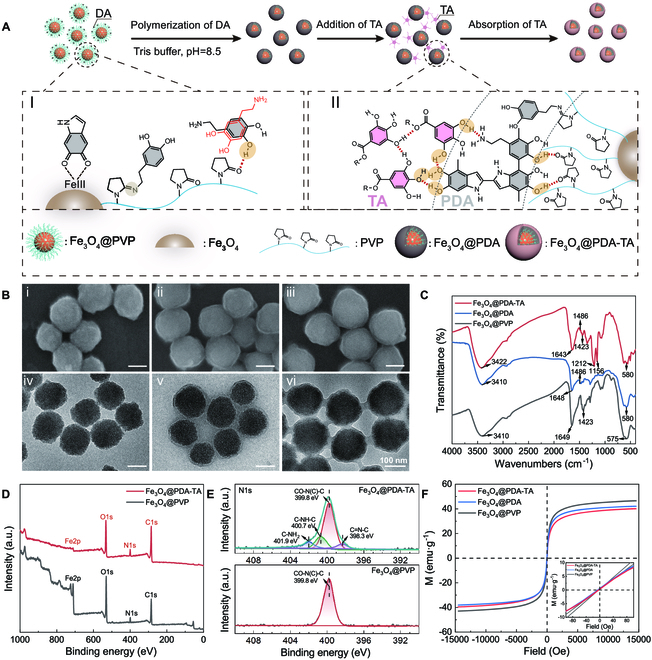
Fabrication and characterization of Fe_3_O_4_@PDA-TA NPs. (A) Schematic illustration of the fabrication process of Fe_3_O_4_@PDA-TA NPs via the in situ polymerization reaction of DA and the further surface modification by TA. (B) SEM (upper row) and TEM (lower row) images of Fe_3_O_4_@PVP (i and iv), Fe_3_O_4_@PDA (ii and v), and Fe_3_O_4_@PDA-TA NPs (iii and vi). (C) FT-IR, (D) wide-spectrum XPS, and (E) high-resolution of N 1s spectra. (F) Magnetic hysteresis loops of Fe_3_O_4_@PVP, Fe_3_O_4_@PDA, and Fe_3_O_4_@PDA-TA NPs. The inset is the enlarged hysteresis loops verifying their superparamagnetism.

The Fe_3_O_4_@PDA-TA NPs, as building blocks of the nanorobots, were fabricated via a simple 2-step method (Fig. 2A). The first step was to coat a PDA shell on Fe_3_O_4_@polyvinyl pyrrolidone (Fe_3_O_4_@PVP) NPs via in situ polymerization of dopamine (DA) molecules concentrated around the core, relying on the complexation between Fe (III) on the Fe_3_O_4_ core and the catechol groups of DA, along with hydrogen bonding between the carbonyl groups of PVP and the phenolic hydroxyl groups of DA (inset I in Fig. 2A). The second step involved surface modification of the intermediate Fe_3_O_4_@PDA NPs with TA to create Fe_3_O_4_@PDA-TA NPs utilizing π–π stacking and hydrogen bonding interactions between TA and the PDA layer (inset II in Fig. 2A) [20,50]. Figure 2B shows scanning electron microscopy (SEM) and transmission electron microscopy (TEM) images of Fe_3_O_4_@PVP, Fe_3_O_4_@PDA, and Fe_3_O_4_@PDA-TA NPs. Compared with the pristine Fe_3_O_4_@PVP NPs (Fig. 2Bi and iv), Fe_3_O_4_@PDA NPs have a smoother surface and an obvious core-shell structure, suggesting that a complete shell was formed on the Fe_3_O_4_ core (Fig. 2Bii and v). On the other hand, the obtained Fe_3_O_4_@PDA-TA NPs share a similar morphology and core-shell structure with Fe_3_O_4_@PDA NPs (Fig. 2Biii and vi). The successful coating of the PDA shell on Fe_3_O_4_@PVP NPs was confirmed by the emerging characteristic absorption band of the benzene ring skeleton at 1,486 cm^−1^ in the Fourier transform infrared (FT-IR) spectrum of Fe_3_O_4_@PDA NPs. In addition, the successful modification of TA onto Fe_3_O_4_@PDA NPs can be verified by the newly formed asymmetric stretching vibration (ν^as^) and symmetric stretching vibration (ν^s^) of C–O–C bonds at 1,212 and 1,156 cm^−1^, respectively, in the FT-IR spectrum of Fe_3_O_4_@PDA-TA NPs (Fig. 2C). Zeta potential (*ζ*) tests revealed that Fe_3_O_4_@PDA-TA NPs gained an enhanced electronegativity (*ζ* = −25.40 mV) after PDA coating and TA modification, attributed to the abundant surface phenolic hydroxyl groups (Fig. S1A). Thermogravimetry (TG) analysis confirmed that the mass content of PDA and TA in Fe_3_O_4_@PDA-TA NPs were 8.8 and 1.35 wt %, respectively (Fig. S1B). The statistical results indicated that, due to the PDA coating and TA modification, the average size of Fe_3_O_4_@PVP, Fe_3_O_4_@PDA, and Fe_3_O_4_@PDA-TA NPs gradually increased from 155.8 and 171.4 nm to 179.6 nm in dry state (Fig. S2). When suspended in water, their hydrodynamic sizes further increased to 217.9, 233.7, and 297.9 nm, correspondingly (Fig. S3).

X-ray photoelectron spectroscopy (XPS) spectra (Fig. 2D) and the high-resolution deconvoluted spectrum of N 1s (Fig. 2E), C 1s, and O 1s (Fig. S4) demonstrated the presence of Fe, C, N, and O elements in Fe_3_O_4_@PDA-TA NPs. The deconvoluted spectrum of N 1s in Fig. 2E revealed the C–NH_2_ (401.9 eV), C–NH–C (400.9 eV) and C=N (398.3 eV) bonds in Fe_3_O_4_@PDA-TA NPs, which were attributed to the PDA, and Schiff base reaction between carbonyl (C=O) groups of PVP from uncoated particle and primary amine (–NH_2_) groups of DA, while in Fe_3_O_4_@PVP NPs there only emerged tertiary nitrogen atoms (399.8 eV). Further, the increased peak intensity of C–N (285.9 eV) in the deconvoluted C 1s spectrum (Fig. S4A) and C–O (532.9 eV) in deconvoluted O 1s spectrum (Fig. S4B) and the decreased peak intensity of Fe–O (529.8 eV) (Fig. S4B) verified the increasing organic content of Fe_3_O_4_@PDA-TA NPs compared with Fe_3_O_4_@PVP NPs. In addition, the quantified atom content variation confirmed the enhanced mass ratio of C and O elements compared to the Fe element. For example, the C/Fe ratio elevated from 4.72 to 53.38 and the O/Fe ratio increased from 2.79 to 12.08 (Table S1), in consistence with the sharp reduction in the intensity of Fe 2p peak of Fe_3_O_4_@PDA-TA NPs in Fig. 2D.

The magnetic hysteresis loop (Fig. 2F) reveals a mass saturation magnetization (*M*_s_) of 39.7 emu g^−1^ for Fe_3_O_4_@PDA-TA NPs, which is slightly lower than pristine Fe_3_O_4_@PVP NPs (44.8 emu g^−1^) and the intermediate product of Fe_3_O_4_@PDA NPs (40.1 emu g^−1^). In addition, the negligible remanent magnetization and coercivity (inset in Fig. 2F) suggest that Fe_3_O_4_@PVP, Fe_3_O_4_@PDA, and Fe_3_O_4_@PDA-TA NPs all possessed a superparamagnetic property. Like Fe_3_O_4_@PVP NPs, the prepared Fe_3_O_4_@PDA and Fe_3_O_4_@PDA-TA colloidal NPs can self-assemble into periodic nanochains exhibiting bright structural colors [51], suggesting that they had monodispersed size and robust interparticle repulsion preventing them from clumping under the magnetic field (Fig. S5). With the decreasing magnetic field strength, the structural color (Fig. S5A) and reflection peaks (Fig. S5B) of aqueous suspensions of Fe_3_O_4_@PVP, Fe_3_O_4_@PDA, and Fe_3_O_4_@PDA-TA NPs redshift gradually due to the increasing interparticle distance (i.e., lattice constant).

### Magnetically driven swarming motions

**Fig. 3.**
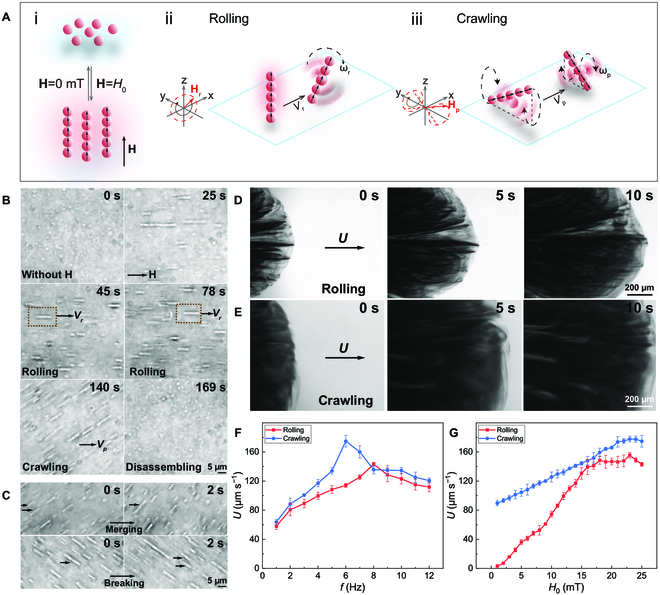
Magnetically driven motions of single Fe_3_O_4_@PDA-TA NRs and collective motions of swarming Fe_3_O_4_@PDA-TA NRs. (A) Schematic illustration and (B) time-lapse microscopic images of the reversible assembly of Fe_3_O_4_@PDA-TA NPs under a static magnetic field H (i), and the rolling (ii) and crawling (iii) moving modes of Fe_3_O_4_@PDA-TA NRs, driven respectively by a rotating magnetic field H_r_(*t*) and a precessing magnetic field H_p_(*t*). (C) Dynamic merging and breaking of Fe_3_O_4_@PDA-TA NRs during propulsion. The* H*_0_ in (B) and (C) is 15 mT, and *f* is 1 Hz. Time-lapse bright-field microscopic images of the swarming Fe_3_O_4_@PDA-TA NRs under (D) H_r_(*t*) and (E) H_p_(*t*), respectively. The *H*_0_ in (D) and (E) is 25 mT, and *f* is 8 Hz and 6 Hz, respectively. Collective velocity (*U*) of the swarm as a function of the (F) frequency (*f)* and (G) strength (*H*_0_) of the applied H_r_(*t*) (red curve) and H_p_(*t*) (blue curve), respectively. Each error bar was obtained from the standard deviation of the velocity derived from 5 swarms.

As is known, superparamagnetic nanoparticles can easily be magnetized and self-assemble into nanoparticle chains due to the dipole–dipole interactions along a static magnetic field **H** (Fig. 3Ai). When a rotating magnetic field **H**_r_(*t*) (Eq. 1) was applied, the self-assembled nanochains experienced a magnetic torque and were forced to rotate around its short axis, while 2 ends of the nanochains rotated around the precession axis when a precessing magnetic field **H**_p_(*t*) (Eq. 2) was applied [33,52].

$$H_{r}\left(t\right)=H_{0}\left[\text{cos}\left(\text{2π}\text{ft}\right)e_{x}+\text{sin}\left(\text{2π}\text{ft}\right)e_{z}\right]$$ (1)

$$H_{p}\left(t\right)=H_{0}e_{y}+H_{0}\left[\text{cos}\left(\text{2π}\text{ft}\right)e_{x}+\text{sin}\left(\text{2π}\text{ft}\right)e_{z}\right]$$ (2)

Here, *H*_0_ is the amplitude of the rotating and precessing magnetic field, respectively. *f* is the alternating frequency; *t* denotes the time; and **e***_x_*, **e***_y_*, and **e***_z_* are the unit vector along the *x*, *y*, and *z* axes, respectively. When the nanochains were rotating or precessing near a substrate, the top and bottom of the nanochains experienced different viscous fluidic drag forces because of local shear gradients. As a result, the hydrodynamic symmetry of the rotating or precessing nanochains was broken, and they can thus perform a “rolling” (Fig. 3Aii) or “crawling” translational motion (Fig. 3Aiii) [48]. Further, the “rolling” and “crawling” nanochains can generate a small local vortex with a flow velocity decaying as *r*^−2^, where *r* is the distance to its rotation axis. When they were in a crowded state, neighboring rollers were attracted and advected by one another via hydrodynamic coupling and further gathered into large compact swarms rotating around their center of mass and translating along the substrate [39,49].

The magnetic assembly, rolling, and crawling motions of single Fe_3_O_4_@PDA-TA NRs are shown in Fig. 3B and Movie S1. Under a static magnetic field (15 mT), dispersed Fe_3_O_4_@PDA-TA NPs assemble into nanoparticle chains with a wide length range from 6.0 to 20.2 μm (Fig. 3B, 0 s–25 s). Despite serious disturbance from Brownian randomization, these nanochains (i.e., single Fe_3_O_4_@PDA-TA NRs) can perform a rolling motion near the substrate when **H**_r_(*t*) was applied (Fig. 3B, 45 s–78 s) and further move in a crawling mode under **H**_p_(*t*) (Fig. 3B, 140 s). Notably, the nanochains dynamically merge and break when moving (Fig. 3C), and can disassemble completely back into dispersed nanoparticles upon the cessation of the magnetic field (Fig. 3B, 169 s). At a high number density, Fe_3_O_4_@PDA-TA NPs can form into cohesive swarms that continuously flow forward in rolling and crawling modes under **H**_r_(*t*) and **H**_p_(*t*), respectively (Fig. 3D and E and Movie S2). Nonetheless, the formed swarms present an obvious difference in collective structures. Specifically, the rolling Fe_3_O_4_@PDA-TA NRs formed into a swarm with clear density variations, featuring multiple dark stripes (or standing vortex) with concentrated nanorobots, interspersed with lighter regions exhibiting sparser distributions (Fig. 3D). In contrast, the crawling nanorobots assembled into a cloud-like swarm covering a larger area with less variation in density distribution (Fig. 3E). Therefore, during task execution, the rolling swarms may be activated to navigate through narrow channels, and the crawling motion mode was subsequently engaged as they approached the destination, enabling the swarm to swiftly and efficiently achieve broad coverage of the targeted area, facilitating subsequent therapeutic tasks.

Manipulating the applied **H**_r_(*t*) or **H**_p_(*t*) can adjust the collective motions of swarming Fe_3_O_4_@PDA-TA NRs. As depicted in Fig. 3F, when the magnetic field strength (*H*_0_) was kept at 25 mT, the collective velocity (*U*) of the nanorobots adopting whether a rolling or crawling motion mode under **H**_r_(*t*) (red line in Fig. 3F) or **H**_p_(*t*) (blue line in Fig. 3F) shows an increasing and then decreasing trend with the increasing *f* from 1 to 12 Hz. This is because the angular velocity *ω* of the rolling or precessing nanorobots gradually increased with the increasing *f*, allowing them to move more quickly on the substrate and create a stronger flow field. When *f* exceeded the critical values, which was the step-out frequency (*f*_c_), the rolling or precessing nanochains in the swarms gradually desynchronize with the external **H**_r_(*t*) or **H**_p_(*t*), resulting in a gradual decrease in *U*. The results in Fig. 3F suggest that the rolling swarm reaches its peak velocity of 142 μm s^−1^ at an *f*_c_ of 8 Hz, and the crawling swarm shows a maximum *U* of about 174 μm s^−1^ at an *f*_c_ of 6 Hz. Additionally, the effect of *H*_0_ on *U* of the swarms was also investigated (Fig. 3G). For the swarming Fe_3_O_4_@PDA-TA NRs in a rolling mode, their collective translational velocity *U* at a fixed *f* of 8 Hz increased with the increasing *H*_0_ until a critical strength of 18 mT was reached (red curve in Fig. 3G). When *H*_0_ exceeded this critical strength, *U* almost stabilized at ~150 μm s^−1^, indicating that the nanorobots overcame the viscous drag from the fluid and rotated synchronously with the applied **H**_r_(*t*). Similarly, *U* of crawling Fe_3_O_4_@PDA-TA NRs swarm under **H**_p_(*t*) with a fixed *f* of 6 Hz increased with the increasing *H*_0_ and reached its maximum of 178 μm s^−1^ when *H*_0_ exceeded 23 mT (blue curve in Fig. 3G). The above results indicate that the crawling swarm had a higher maximum mobility than the rolling one. This difference can be attributed to the less moment of inertia in the “crawling” mode, leading to the less energy required for the precessing nanorobots to rotate and move in the liquid medium. Under dark-field microscopy, the flowing swarm of Fe_3_O_4_@PDA-TA NRs displayed a bright red structural color (Fig. S6 and Movie S3). This observation confirmed their high stability during collective motion, as random aggregation would disrupt the ordered arrangement necessary for this structural color to appear.

### Targeted photothermal bacterial elimination

**Fig. 4.**
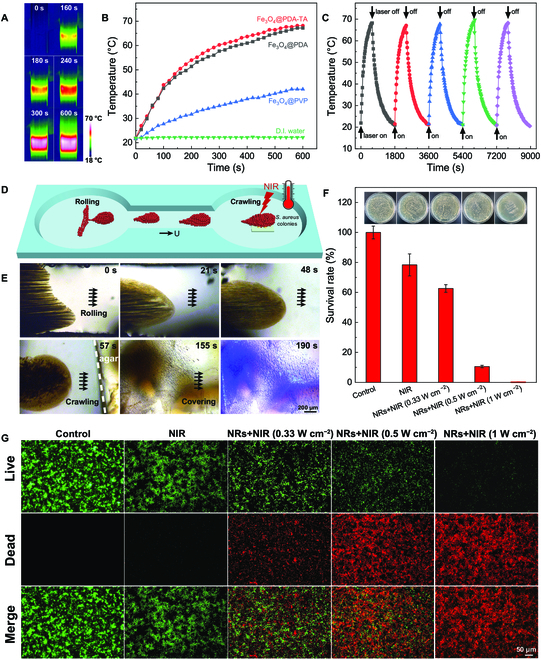
Targeted photothermal eradiation of bacteria on a superficial site by swarming Fe_3_O_4_@PDA-TA NRs. (A) Infrared thermal images of the aqueous suspension of Fe_3_O_4_@PDA-TA NPs when irradiated by a NIR laser (808 nm). (B) Photothermal heating curves of the aqueous suspensions of Fe_3_O_4_@PVP, Fe_3_O_4_@PDA, and Fe_3_O_4_@PDA-TA NPs. (C) Cyclic photothermal heating curve of the aqueous suspension of Fe_3_O_4_@PDA-TA NPs. The nanoparticle concentration *C*_p_ and the laser power density *I* in (A) to (C) are set at 250 μg ml^−1^ and 1.0 W cm^−2^, respectively. (D) Schematic illustration and (E) time-lapse microscopic images of the targeted movement and photothermal treatment of swarming Fe_3_O_4_@PDA-TA NRs toward a simulated superficial infection site. The* H*_0_ is 20 mT, and *f* is 3 Hz. (F) Survival rates of *S. aureus* after being treated by Fe_3_O_4_@PDA-TA NPs (250 μg ml^−1^) under NIR irradiation with an *I* of 0.33, 0.5, and 1.0 W cm^−2^, respectively. The insets are digital photographs of *S. aureus* colonies on agar plates. The *S. aureus* bacteria treated with DI water and only laser irradiation without Fe_3_O_4_@PDA-TA NRs are depicted as the control and NIR group, respectively. (G) Fluorescence microscopic images of the *S. aureus* bacteria treated at different conditions after live/dead staining.

To explore the potential of the nanorobots in the superficial photothermal bacterial elimination, the light absorption properties and photothermal effect of Fe_3_O_4_@PDA-TA NPs were tested at first. As shown in Fig. S7A, Fe_3_O_4_@PVP, Fe_3_O_4_@PDA, and Fe_3_O_4_@PDA-TA NPs all have a high dispersity in water even after the application of a high-strength **H** (1,000 Gs). Notably, the Fe_3_O_4_@PDA NP and Fe_3_O_4_@PDA-TA NP suspensions (125 μg ml^−1^) exhibit a darker color (Fig. S7A) and higher light absorption at 808 nm (Fig. S7B) compared to Fe_3_O_4_@PVP NPs. Further, the minimal difference in light absorption between Fe_3_O_4_@PDA and Fe_3_O_4_@PDA-TA suspensions suggests that PDA primarily contributes to enhanced light absorption, with minimal influence from the TA modification. The photothermal conversion ability of Fe_3_O_4_@PDA-TA, Fe_3_O_4_@PDA, and Fe_3_O_4_@PVP NPs was further investigated, as indicated by Fig. S7C and D and Table S2. The photothermal conversion efficiency (*η*) of Fe_3_O_4_@PDA and Fe_3_O_4_@PDA-TA NPs increased to 42.0% and 42.5%, respectively, compared to18.1% for Fe_3_O_4_@PVP NPs. As recorded by the infrared thermography (Fig. 4A), the temperature of Fe_3_O_4_@PDA-TA NPs aqueous suspension rapidly increased up to 68.2°C after irradiation for 600 s with an 808-nm laser at a power density (*I*) of 1.0 W cm^−2^. The photothermal heating curves indicate that, under the same NIR irradiation conditions, different from the negligible temperature rise of the deionized (DI) water (control group), the Fe_3_O_4_@PVP NP suspension was heated from 22 to 42°C owing to photothermal conversion of the Fe_3_O_4_ component [53]. Notably, the Fe_3_O_4_@PDA and Fe_3_O_4_@PDA-TA NP suspensions exhibit nearly identical heating curves, reaching even higher temperatures of 67.2 and 68.2°C within 600 s, respectively (Fig. 4B). This result suggests that Fe_3_O_4_@PDA-TA NPs exhibited a strong photothermal effect, and their photothermal conversion mainly came from the combined contributions of the PDA shell and the inner Fe_3_O_4_ core. In addition, Fe_3_O_4_@PDA-TA NPs demonstrate excellent repeatability and stability in photothermal conversions, as evidenced by their cycling photothermal heating–cooling curves in Fig. 4C.

The influence of NIR laser power density *I* and nanoparticle concentration (*C*_p_) on the photothermal conversion of Fe_3_O_4_@PDA-TA NPs was further explored (Fig. S8). As shown in Fig. S8A, the increasing *I* results in steeper photothermal heating curves, and the temperature of the aqueous suspension of Fe_3_O_4_@PDA-TA NPs (250 μg ml^−1^) reaches 43.7, 49.6, 68.2, 74.7, and 85.8°C after NIR irradiation for 600 s at an *I* of 0.33, 0.5, 1.0, 1.5, and 2.0 W cm^−2^, respectively. Similarly, the increasing *C*_p_ leads to a higher heat rate of the aqueous Fe_3_O_4_@PDA-TA NP suspension under NIR irradiation (*I* = 1.0 W cm^−2^). For instance, it only took 100 and 80 s for the Fe_3_O_4_@PDA-TA NP suspension to reach a temperature higher than 50°C when *C*_p_ was 500 and 1,000 μg ml^−1^, respectively. In addition, with *C*_p_ increasing from 125, 250, and 500 to 1,000 μg ml^−1^, the final temperature of the aqueous suspensions after the NIR irradiation for 600 s rose from 56.7, 68.2, and 77.8 to 79.3°C, respectively (Fig. S8B). Notably, the optimal conditions for Fe_3_O_4_@PDA-TA NRs to perform photothermal bacterial elimination were found to be at a *C*_p_ of 250 μg ml^−1^ and an *I* below 1.0 W cm^−2^, as the final temperature of the suspension is in a mild hyperthermia range (<70°C), which can reduce the potential cytotoxicity of high-concentration nanoparticles and the hyperthermia damage of intense laser irradiation to normal tissues. Interestingly, magnetically assembled Fe_3_O_4_@PDA-TA NPs exhibited superior photothermal performance compared to their dispersed counterparts (Fig. S9). Suspensions containing assembled Fe_3_O_4_@PDA-TA NPs displayed consistently higher heating rates under a magnetic field of 200 Gs across a wide range of *C*_p_ from 125 to 1,000 μg ml^−1^, compared to their dispersed counterparts. This enhanced photothermal effect resulted in a final temperature difference of 5.5, 3.2, 1.6, and 1.2°C between the assembled and dispersed suspensions within 600 s at *C*_p_ of 125, 250, 500, and 1,000 μg ml^−1^, respectively. These findings suggest that the condensed local particle density achieved through magnetic assembly enhances the photothermal heating capacity, particularly at lower concentrations.

Benefitting from high photothermal performance and swarming motions, Fe_3_O_4_@PDA-TA NRs are envisioned to realize photothermal elimination of bacteria in a motile-targeting manner. To verify this, we used a piece of agar gel with inoculated bacteria *Staphylococcus aureus* colonies on its surface as a simulated superficial infection site, and the agar gel was put in the right pool of a microfluidic channel to serve as the target (Fig. 4D). For effective photothermal bacterial elimination at a remote superficial infection site, the swarming Fe_3_O_4_@PDA-TA NRs need to achieve wide-range coverage of the site from a distance before performing efficient elimination. When driven by **H**_r_(*t*) with *H*_0_ of 20 mT and an *f* of 3 Hz, a swarm of Fe_3_O_4_@PDA-TA NRs was activated to pass through the middle canal, and reached the right pool with the target in a rolling mode (0 to 48 s in Fig. 4E). Upon arriving at the right pool, the Fe_3_O_4_@PDA-TA swarm was adjusted into a relatively widespread state (i.e., crawling mode) by switching the driving magnetic field from a rolling **H**_r_(*t*) to a precessing **H**_p_(*t*). In this crawling mode, the swarming Fe_3_O_4_@PDA-TA NRs could climb up the bacterial-inoculated agar gel target and cover the bacteria colonies (57 to 155 s in Fig. 4E). Then, a beam of NIR light was locally applied to activate the photothermal conversion of Fe_3_O_4_@PDA-TA NRs, generating hyperthermia to eradicate bacteria in the target area (corresponding to 190 s in Fig. 4E and Movie S4).

To investigate the antibacterial effect of Fe_3_O_4_@PDA-TA NRs in photothermal treatment, *S. aureus* bacteria were subjected to different experimental conditions, including pure NIR light exposure (1.0 W cm^−2^, NIR group), the employment of Fe_3_O_4_@PDA-TA NRs (*C*_p_ = 250 μg ml^−1^), and NIR irradiation at different *I* of 0.33, 0.5, and 1.0 W cm^−2^ (NRs + NIR groups). In the NRs + NIR groups, Fe_3_O_4_@PDA-TA NPs were mixed with *S. aureus* bacteria before being irradiated by the NIR laser at different *I* for 10 min, and then incubated on agar plate for colony-forming unit (CFU) counts. Meanwhile, the antibacterial experiment without nanoparticles or NIR irradiation was used as a control group. The results are shown in Fig. 4F. The agar plate is almost covered with *S. aureus* colonies in the NIR group, and the survival rate reaches 78.4%. In contrast, for the bacteria treated by Fe_3_O_4_@PDA-TA NPs and NIR irradiation, the numbers of *S. aureus* colonies observed on the agar plate reduce with the increasing *I*, and the survival rate decrease to 62.6%, 10.6%, and even 0.2% accompanying with an *I* increased from 0.33 and 0.5 to 1.0 W cm^−2^, respectively. The good photothermal sterilization capacity of swarming Fe_3_O_4_@PDA-TA NRs can also be verified by the fluorescence images of *S. aureus* under different experimental conditions (Fig. 4G). In order to distinguish, the live *S. aureus* bacteria were dyed green, and the dead one was dyed red. *S. aureus* kept alive and almost no dead *S. aureus* were found in the control group and the NIR group, indicating that the NIR irradiation alone had a minimal capacity to cause *S. aureus* apoptosis. In contrast, dead *S. aureus* bacteria were observed in NRs + NIR groups even at a weak *I* of 0.33 W cm^−2^. When *I* was increased to 0.5 W cm^−2^, most *S. aureus* were dead, and especially, almost no alive *S. aureus* appeared in the NRs + NIR group with an *I* of 1.0 W cm^−2^. The above results indicate that Fe_3_O_4_@PDA-TA NRs show a great potential to cover a targeted infection area from a distance via their controllable swarming motions and then rapidly eradicate bacteria residing on the infection site in virtue of their high photothermal performance.

### Targeted chemical bacterial elimination

**Fig. 5.**
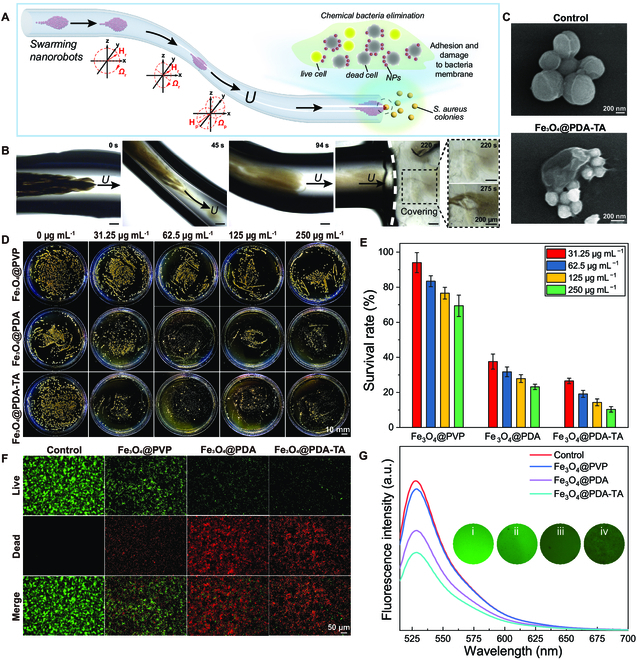
Targeted chemical elimination of bacteria in a deep-seated site by swarming Fe_3_O_4_@PDA-TA NRs. (A) Schematic illustration and (B) time-lapse microscopic images of swarming Fe_3_O_4_@PDA-TA NRs targeting a simulated deep-seated infection site by navigating through a zigzag narrow microtube. *H*_0_ was 20 mT, and *f* was 3 Hz. (C) SEM images of *S. aureus* bacteria before and after treatment with Fe_3_O_4_@PDA-TA NPs. (D) Photographs of the bacteria colonies on agar plates and (E) survival rates of *S. aureus* after being treated with Fe_3_O_4_@PVP, Fe_3_O_4_@PDA, and Fe_3_O_4_@PDA-TA NPs at different *C*_p_. (F) Fluorescence images of *S. aureus* bacteria after being treated with Fe_3_O_4_@PVP, Fe_3_O_4_@PDA, and Fe_3_O_4_@PDA-TA NPs and DI water (Control). (G) Fluorescence intensities and the fluorescence images of *S. aureus* bacteria stained by Rhodamine123 after cocultured with DI water (Control) (i), Fe_3_O_4_@PVP (ii), Fe_3_O_4_@PDA (iii), and Fe_3_O_4_@PDA-TA (iv) NPs. The *C*_p_ of Fe_3_O_4_@PVP, Fe_3_O_4_@PDA, and Fe_3_O_4_@PDA-TA NP used in (F) and (G) is 250 μg ml^−1^.

Besides the capacity to execute superficial photothermal bacterial elimination, Fe_3_O_4_@PDA-TA NRs show great potential to actively eliminate bacteria located within deep-seated infection sites utilizing their chemical antibacterial effects. For nanorobots to eliminate bacteria in a deep-seated site, they must first navigate through tortuous and narrow passages to reach the target site. If NIR light cannot be directly applied from all angles or delivered through a fiber optic source at these deep locations, the nanorobots must rely solely on their inherent chemical properties to kill bacteria in these areas. To verify this, a deep-seated infection site, simulated by a piece of agar gel with grown *S. aureus* colonies, was placed at the far end of a zigzag microtube with a narrow neck (inner diameter: ~400 μm) (Fig. 5A). As demonstrated by Fig. 5B and Movie S5, the swarming Fe_3_O_4_@PDA-TA NRs can smoothly traverse the zigzag microtube under the navigation of the rotating **H**_r_(*t*) with adjustable orientations (0 to 220 s in Fig. 5B). Upon approaching the target, the rotating **H**_r_(*t*) was switched into a precessing **H**_p_(*t*) (220 s in Fig. 5B) and Fe_3_O_4_@PDA-TA NRs can further delve deeper into the target through branching narrow ravines (down to 30 μm wide) and gradually distribute themselves around the bacterial colonies (220 to 275 s in Fig. 5B). This result suggests that the swarming Fe_3_O_4_@PDA-TA NRs can travel a long distance and transverse narrow passages with varying sizes to target the deep-seated bacteria, laying the groundwork for deep antibacterial treatment.

The chemical antibacterial activity of Fe_3_O_4_@PDA-TA NRs primarily originates from their surface TA molecules. Specifically, due to abundant phenolic hydroxyl groups from surface TA molecules, Fe_3_O_4_@PDA-TA NPs (i.e., building blocks of nanorobots) exhibit excellent adhesion to the bacteria membrane owing to their easy association with the peptidoglycan, lipopolysaccharide, teichoic acid, protein, and lipid on the bacterial membrane [54]. Upon membrane adhesion, Fe_3_O_4_@PDA-TA NPs can cause cell membrane destruction and exudation of cell contents [55]. As verified by Fig. 5C, in contrast to smooth and spherical normal *S. aureus* cells (control group), the cell with attached Fe_3_O_4_@PDA-TA NPs displayed collapsed and wrinkled surface morphology. To evaluate the chemical antibacterial efficacy, the dose-dependent cytotoxicity of Fe_3_O_4_@PDA-TA NPs to *S. aureus* cells was quantitatively evaluated (Fig. 5D and E). As expected, the viability of *S. aureus* cells remained relatively high (>69%) when exposed to Fe_3_O_4_@PVP NPs over a wide range of concentrations. In contrast, the cell viability decreased from 26.5% to 10.3% as the concentration of Fe_3_O_4_@PDA-TA NPs increased from 31.25 to 250 μg ml^−1^. Surprisingly, Fe_3_O_4_@PDA NPs also have excellent chemical antibacterial efficacy. This is attributed to the abundant hydroxyl groups on the surfaces of both nanoparticles, likely leading to a shared contact-active antibacterial mechanism [56]. The Fe_3_O_4_@PDA-TA NPs have a higher antibacterial efficacy than Fe_3_O_4_@PDA NPs at equivalent concentrations (e.g., 10.3% versus 23.0% cell viability at 250 μg ml^−1^), although the former may experience partial detachment of surface-bound TA molecules (29.4% within 12 h) (Fig. S10). The observed mild antibacterial effect of Fe_3_O_4_@PVP NPs may come from the release of Fe^2+^ ions that generate ROS in the bacterial environment [42,57]. The high chemical antibacterial efficacy of Fe_3_O_4_@PDA-TA NPs was also confirmed by fluorescence microscopic images of dead (red) and surviving (green) *S. aureus* cells after treatment (Fig. 5F). Due to the high chemical antibacterial activity of Fe_3_O_4_@PDA and Fe_3_O_4_@PDA-TA NPs, most *S. aureus* cells were killed within 24 h. In contrast, minimal cell death was observed when the *S. aureus* cells were exposed to Fe_3_O_4_@PVP NPs and no cell death was detected with DI water (Control group). To further verify the antibacterial mechanism, the bacteria after different treatments were stained using the cell membrane permeability indicator Rhodanmine123, of which the fluorescence intensity is positively correlated with membrane potential and inversely correlated with the membrane permeability [58]. The Fe_3_O_4_@PDA-TA group exhibited the weakest fluorescence intensity, indicating that Fe_3_O_4_@PDA-TA NPs were most effective at disrupting the cell membranes of *S. aureus* bacteria compared to Fe_3_O_4_@PVP and Fe_3_O_4_@PDA NPs (Fig. 5G). These results revealed that the swarming Fe_3_O_4_@PDA-TA NRs can actively target bacteria in remote and narrow deep-seated sites and exert sustained chemical antibacterial effects. With combined antibiotic-free photothermal and chemical antibacterial activities, Fe_3_O_4_@PDA-TA NRs show great potential to treat complicated infection lesions involving both superficial and deep-seated infection sites.

## Discussion

Bacterial infections that spread from the surface to deep sites are a frequent complication in various situations, including surgical sites, traumatic wounds, deep burns, and lung infections [59,60]. For example, in ventilator-associated pneumonia (VAP), a prevalent complication in intensive care unit (ICU) patients, bacteria can infiltrate the lower respiratory tract, spreading from the bronchi and bronchioles to the alveoli. Systemic antibiotic administration is occasionally proved inefficient or ineffective, particularly against MDR and extensively drug-resistant (XDR) bacteria. Benefitting from their small sizes and motility, MNRs are expected to deliver various antibacterial agents or energies to many hard-to-reach infection sites with a high targeting efficiency, and thus may provide revolutionary changes to infection treatment [39,41–45]. However, it is of great challenge for the so-far developed MNRs to effectively manage complicated superficial-to-deep bacterial infections. In this work, we demonstrate that the swarming Fe_3_O_4_@PDA-TA NRs incorporated with photothermal and natural antibacterial agents can perform targeted bacteria elimination in complicated bacterial infection scenarios involving both superficial and deep-seated sites.

The swarming magnetic nanorobots with integrated superficial photothermal and deep chemical strategies for targeted bacterial elimination represent a significant advancement in the field of nanomedicine and micro/nanorobotics. At first, the swarming behavior of Fe_3_O_4_@PDA-TA NRs offers distinct advantages for targeted delivery and distribution within complex infection environments. Their rolling and crawling collective motions enable them to cohesively approach and rapidly achieve broad coverage of a targeted superficial area in open spaces. This facilitates subsequent uniform photothermal energy distribution and efficient eradication of surface-residing bacteria across a wide area. When facing a deep-seated infection scenario, they can navigate through confined, narrow, and tortuous passages, and actively concentrate at the deep infection site to enhance the overall efficacy of the treatment while minimizing potential off-target effects. In stark contrast, passive antibacterial nanoagents and single MNRs generally suffer from limited target coverage and slow accumulation time at the superficial infection sites, and also cannot effectively penetrate into deep-seated infection sites for bacterial eradication [46,61]. Second, by incorporating photothermal and natural antibacterial agents, the swarming Fe_3_O_4_@PDA-TA NRs can effectively combat bacterial infections with high precision, enhanced efficiency, and task flexibility. Specifically, together with wide surface coverage and deep penetration through narrow cavities, the swarming Fe_3_O_4_@PDA-TA NRs not only can rapidly eradicate bacteria residing on the superficial infection site through localized photothermal ablation but also exert sustained antibacterial action in the deep infection site through their inherent chemical activities. In contrast, those magnetic MNRs relying on single-mode physical (e.g., mechanical force and photothermal hyperthermia) or chemical antibacterial strategy (e.g., antibiotic drugs and ROS/nitric oxide generation) usually suffer from limited applicability or low bacterial elimination efficiency [43,62–64]. While some studies have reported MNRs integrating physical and chemical sterilization, they typically involve large microrobots (tens of micrometers) with catalytic properties or loaded antibiotics [42,65,66]. These approaches often require external chemicals (e.g., H_2_O_2_), have limited access to narrow cavities, raise biosafety concerns, and may be susceptible to AMR.

Several challenges and limitations must be addressed to fully realize the clinical potential of this technology. A crucial aspect is ensuring the in vivo biocompatibility and safety of Fe_3_O_4_@PDA-TA NRs. While their constituent materials (Fe_3_O_4_ NPs, PDA, and TA) have been reported to possess biocompatibility [67,68], further investigations are necessary to evaluate their biodegradability, long-term effects, and potential immunogenicity within biological systems. Additionally, further improvements in the antibacterial efficacy of Fe_3_O_4_@PDA-TA NRs are desirable. Potential strategies include incorporating additional functionalities into the building blocks, such as extra chemotactic engines to allow autonomous bacterial targeting, surface nanospikes to facilitate membrane disruption, or co-encapsulation of antibacterial agents (e.g., antibiotics, AMPs, enzymes, or immunomodulatory drugs) [69–71]. Envisioning widespread clinical use, future endeavors should prioritize the scalability and cost-effectiveness of Fe_3_O_4_@PDA-TA NPs production methods. Additionally, it is of immense promise to expand the functionalities of these swarming magnetic nanorobots for broader therapeutic applications beyond bacterial elimination. For instance, incorporating antitumor or thermolytic drugs to the building blocks of the nanorobots could enable them to realize multimodal tumor therapies and thrombosis dissolution [48,72].

In summary, we have designed swarming multifunctional magnetic Fe_3_O_4_@PDA-TA NRs that can achieve superficial photothermal and deep chemical bacterial elimination. The Fe_3_O_4_@PDA-TA NPs (building blocks) are successfully fabricated by introducing a photothermal-conversion PDA layer onto the superparamagnetic Fe_3_O_4_ core and an inherent antibacterial layer of natural product TA on the outermost shell through a simple 2-step method. They show a superparamagnetic property with a high *M*_s_ of 39.7 emu g^−1^ and an efficient photothermal conversion with a *η* of 42.54%. Under navigation of rotating **H**_r_(*t*) and precessing **H**_p_(*t*), Fe_3_O_4_@PDA-TA NPs can assemble into rolling or wobbling nanochains and further self-organize into large energetic microswarms continuously flowing forward with a maximum velocity of 178 μm s^−1^. Thus, the swarming Fe_3_O_4_@PDA-TA NRs can collectively move toward and cover a superficial infection site from a distance, and rapidly eradicate bacteria residing on the surface upon exposure to NIR light due to their efficient photothermal conversion of the Fe_3_O_4_ and PDA components. The Fe_3_O_4_@PDA-TA NRs achieve a high elimination rate of 99.8% against *S. aureus* within 10 min under NIR irradiation. Furthermore, they can also penetrate and enter deep-seated infection sites through narrow and tortuous passages, and exert sustained antibacterial action utilizing their inherent chemical activities mainly based on their surface TA-induced easy cell adhesion and subsequent membrane destruction, leading to sustained antibacterial action (chemical bactericidal rate of 89.7% against *S. aureus* in 24 h). The swarming Fe_3_O_4_@PDA-TA NRs show a great potential to execute targeted bacterial elimination at the superficial and deep-seated infection sites simultaneously or sequentially. This work paves a way for the development of future therapeutic microsystems with multifunctional synergies, task flexibility, and high efficiency, potentially applicable to a broader range of diseases.

## Materials and Methods

### Materials

The superparamagnetic Fe_3_O_4_@PVP NPs were fabricated using our previously reported method [51]. Dopamine hydrochloride (DA-HCl) was purchased from Macklin Biochemical Technology Co. Ltd. (Shanghai, China). Tris (hydroxymethyl) aminomethane hydrochloride buffer (tris-HCl; pH 8.5) and TA (95%) were provided by Aladdin Reagent Co. Ltd. (Shanghai, China). Phosphate-buffered saline (PBS; pH 7.4) was acquired from Thermo Fisher Scientific Inc. (USA). Ethanol [analytical reagent (AR)] and sodium chloride (NaCl, AR) were purchased from Sinopharm Chemical Reagent Co. Ltd. (Shanghai, China). Tryptone [biochemical reagent (BR)] and soy peptone (BR) were obtained from Shuangxuan Factory (Beijing, China). Nutrient agar (BR) was achieved from Qingdao Hope Bio-Technology Co. Ltd (Qingdao, China). Syto 9, propidium iodide (PI), and Rhodanmine123 kit were obtained from Beyotime Biotechnology Inc. (Shanghai, China). DI water (18.20 MΩ cm^−1^) was purified using a Milli-Q system (Millipore, Burlington, MA, USA). All the reagents were used as received without further purification.

### Fabrication of Fe_3_O_4_@PDA-TA NPs

Typically, Fe_3_O_4_@PVP NPs with a diameter of ~150 nm (23.6 mg), DA-HCl (0.33 mg ml^−1^), and 15 ml of tris-HCl buffer solution (pH 8.5) were mixed in a 3-orifice flask by sonification. The above reaction solution was stirred for 1.5 h while being sonicated, and the dispersion turned from dark brown to gray black. The mixture was centrifuged, and the nanoparticles were collected at the bottom, after which the nanoparticles were rinsed with DI water and ethanol 3 times to remove the unreacted monomers and oligomers. Subsequently, the obtained Fe_3_O_4_@PDA NPs (11.8 mg) were redispersed in TA aqueous solution (2.5 mg ml^−1^) and sonicated for 5 min and left to stay overnight. The dispersion was centrifuged and rinsed with DI water and ethanol 3 times to clear off the unabsorbed TA molecules, and Fe_3_O_4_@PDA-TA NPs were stored in DI water for later use.

### Characterization

The morphology and structure of the nanoparticles were characterized by a field-emission SEM (Hitachi S-4800, Japan) and a TEM (JEOL, JEM-2100F, Japan). The XPS of the nanoparticles was obtained from the -ray photoelectron spectrometer (Shimadzu, AXIS SUPRA, Japan) at a voltage of 15 kV and a current of 5 mA in the broad spectra and 10 mA in the fine spectra. The FT-IR spectra were measured by a Fourier transform infrared spectrometer (Thermo Fisher Scientific, Nicolet6700, USA). The TG curves were tested by a TG analyzer (NETZSCH, STA449F3, Germany) in the temperature range from 25 to 800°C with a heating rate of 10°C min^−1^ at air atmosphere. Zeta potentials (*ζ*) and hydrodynamic sizes were characterized by a Zetasizer Nano ZS90 analyzer (Malvern, UK). The ultraviolet (UV)–visible (vis)–NIR absorption spectra measurement were recorded using a UV-vis-NIR spectrophotometer (PerkinElmer, Lambda 750 S, USA). Reflection spectra of nanoparticle dispersions were measured through a fiber optic spectrometer (Ocean Optics, USB2000+, USA). The magnetic hysteresis loops were tested by a vibrating sample magnetometer (LakeShore 7404s, USA) at 20°C.

### Magnetic propulsion of single and swarming Fe_3_O_4_@PDA-TA NRs

The magnetic propulsion experiments were conducted in a customized magnetic field system consisting of electric current supplies (ATA-309 Power Amplifiers, China), a signal source (NI USB-6343, USA), and a 3-axis Helmholtz electromagnetic coil. An aqueous suspension of Fe_3_O_4_@PDA-TA NPs at a certain concentration (*C*_p_ = ~50 μg ml^−1^ for observation of single NRs and *C*_p_ = ~5,000 μg ml^−1^ for observation of swarming nanorobots) was added dropwise to a glass substrate or a microfluidic channel, and then a permanent magnet (3,000 Gs) was used to collect the dispersed nanoparticles and allow them to settle near the substrate. The Fe_3_O_4_@PDA-TA NPs on the substrate were transferred to the coil mounted on an inverted optical microscope (Leica DM3000M, Germany) and then activated and navigated by applying a rotating magnetic field **H**_r_(*t*) or a precessing magnetic field **H**_p_(*t*) with different directions, strength *H*_0_, and frequency *f*. All videos were analyzed using Video Spot Tracker V08.01 and ImageJ software.

### Photothermal performance testing

Aqueous suspensions (1.0 ml) of Fe_3_O_4_@PDA-TA NPs with different concentrations (*C*_p_ = 125, 250, 500, and 1,000 μg ml^−1^) and Fe_3_O_4_@PVP and Fe_3_O_4_@PDA NPs with *C*_p_ of 250 μg ml^−1^ were added to a standard cuvette and exposed to the NIR laser (808 nm, Leize BOT808-2D200F-S2, China) with different power densities (*I*) of 0.33, 0.5, 1.0, 1.5, and 2.0 W cm^−2^ at room temperature (25°C) for 600 s. The temperature of the suspension was recorded at 20-s intervals using a thermography camera (FLIR T420, USA). The UV-vis-NIR absorbance of Fe_3_O_4_@PVP, Fe_3_O_4_@PDA, and Fe_3_O_4_@PDA-TA NP suspensions (*C*_p_ = 125 μg ml^−1^) were tested by a UV-vis-NIR spectrophotometer. Photothermal conversion efficiency (*η*) of the nanoparticles was calculated in the Supplementary Materials.

### Photothermal bacterial elimination

*S. aureus* bacteria (as a model bacterium) were cultured in the tryptic soy broth (TSB) liquid medium (pH 7.2 to 7.4 with 15 g l^−1^ tryptone, 5 g l^−1^ soy peptone, and 5 g l^−1^ NaCl) in a shaking incubator at 37°C for 12 h to obtain bacterial suspension (~1 × 10^9^ CFU ml^−1^). Prior to use, all materials underwent sterilization by exposure to UV light. The targeted photothermal elimination of *S. aureus* bacteria was conducted within a microfluidic chip with 2 open reservoirs interconnected by a narrow canal. A piece of agar gel inoculated with *S. aureus* colonies was placed at the right reservoir of the microchannel as a simulated superficial infection site, and the swarming Fe_3_O_4_@PDA-TA NRs were navigated to cross the canal under **H**_r_(*t*) (*H*_0_ = 20 mT, *f* = 3 Hz) and then cover the targeted bacteria colonies under **H**_p_(*t*) (*H*_0_ = 20 mT, *f* = 3 Hz). After coverage, the nanorobots were irradiated by a NIR laser for 10 min to eliminate the bacteria. To evaluate photothermal bacterial elimination at different *I*, the aqueous dispersions of Fe_3_O_4_@PDA-TA NPs were mixed with bacterial suspension (1 × 10^9^ CFU ml^−1^) in a 24-well plate to form a 1-ml mixture with a *C*_p_ of 250 μg ml^−1^ and followed by irradiation with 808-nm NIR light (0.33, 0.5, and 1.0 W cm^−2^) for 10 min. The bacteria mixed with DI water and treated without NIR laser, and mixed with DI water and irradiated by NIR laser were set as the control and NIR group, respectively. Bacterial survival rate was then evaluated by the standard agar plate counting method. Briefly, the suspensions were diluted with sterile PBS buffer (pH 7.4, 0.01 M) and spread on a petri dish containing the agar plates to culture for another 12 h at 37°C in an illumination incubator (Hengzi, SPX-150-GBH, China). The number of colonies and bacterial survival rates were calculated. Bacterial survival rate (%) = (number of bacteria colonies in experimental group/number of viable bacteria colonies in control group) × 100%. The tests of each experiment were replicated 3 times. The live/dead staining test was also carried out to visualize living and dead bacteria. Specially, the liquid medium of each group in the 24-well plate was removed, and the bottom bacteria were retained. Dye solution (500 μl) with 5 μM Syto 9 and 5 μM PI was added. After 15 min, the living and dead bacteria in each cell of the 24-well plate were captured by an inverted fluorescence microscope (Leica DM3000B, Germany) (green fluorescence for live bacteria and red fluorescence for dead bacteria).

### Chemical bacterial elimination

For targeted chemical bacteria elimination, a piece of agar gel inoculated with *S. aureus* colonies was placed at the far end of a zigzag microtube to serve as a simulated deep-seated infection site. The swarming Fe_3_O_4_@PDA-TA NRs were navigated to cross the microtube under **H**_r_(*t*) (*H*_0_ = 20 mT, *f* = 3 Hz) and then cover the targeted bacteria colonies under **H**_p_(*t*) (*H*_0_ = 20 mT, *f* = 3 Hz) for subsequent chemical bacterial elimination. To evaluate the dose-dependent cytotoxicity of the nanoparticles to *S. aureus* bacteria, 500 μl of bacterial suspension (1 × 10^9^ CFU ml^−1^) was mixed with aqueous dispersions of Fe_3_O_4_@PVP, Fe_3_O_4_@PDA, and Fe_3_O_4_@PDA-TA NPs in a 24-well plate to prepare 1-ml mixtures with *C*_p_ of 31.25, 62.5, 125, and 250 μg ml^−1^, respectively. The bacterial suspension mixed with 500 μl of DI water was set as the control group. After incubation at 37°C for 24 h, bacterial survival rate was then evaluated by the standard agar plate counting method and the live/dead staining test. In addition, cell membrane permeability of *S. aureus* bacteria after being cocultured with Fe_3_O_4_@PVP, Fe_3_O_4_@PDA, and Fe_3_O_4_@PDA-TA NP suspensions (*C*_p_ =250 μg ml^−1^) and DI water (as control group) was also tested. Briefly, the culture medium in each cell of 24-well plate was removed. Rhodamine 123 solution (500 μl; 10 μM) was added and dyed in the dark for 30 min. The fluorescence microscopic images were captured by an inverted fluorescence microscope (Leica DM3000B, Germany). The fluorescence intensity was measured by a fluorescence spectrophotometer (SHIMADZU, RF-6000, Japan) at an excitation light wavelength of 510 nm.

### Cumulative release of TA

To test the cumulative release of TA molecules, 1.375 ml of the aqueous suspension of freshly prepared Fe_3_O_4_@PDA-TA NPs (8 mg ml^−1^) was shaken at 150 rpm under 37°C in a shaking incubator (Yichun, TS-100C, China). At predetermined time points (1, 2, 3, 5, 7, and 12 h), the suspension was centrifuged to separate the released TA from the nanoparticles. The supernatant was collected, and fresh DI water was added to the remaining pellet to maintain the initial volume. The amount of released TA in the supernatant was quantified by UV-vis-NIR spectroscopy at 280 nm using a Shimadzu UV-2550 spectrophotometer (Japan). The cumulative release percentage of TA was calculated as follows: Cumulative released TA (%) = (amount of released TA/total amount of adsorbed TA) × 100%. The experiments were repeated for 3 times.

## Data Availability

All data needed to support the conclusions of this manuscript are included in the main text or Supplementary Materials. Source data are available in the Supplementary Materials.

## Supplementary Materials

Supplementary 1 Figs. S1 to S10 Table S1 and S2 Movies S1 to S5 Reference
