# Supplementary material for: Swarming Magnetic Fe3O4@Polydopamine-Tannic Acid Nanorobots: Integrating Antibiotic-Free Superficial Photothermal and Deep Chemical Strategies for Targeted Bacterial Elimination
Source: Research (Wash D C). 2024 Jul 31;7:0438. doi: 10.34133/research.0438 (PMC11289052; doi:10.34133/research.0438)
Supplement: Supplementary 1 — Figs. S1 to S10 Table S1 and S2 Movies S1 to S5 Reference [file research.0438.f1.zip › Supporting Information-revised version.docx]

Supplementary Materials for

**Swarming Magnetic Fe_3_O_4_@Polydopamine-Tannic Acid Nanorobots: Integrating Antibiotic-Free Superficial Photothermal and Deep Chemical Strategies for Targeted Bacterial Elimination**

*Luying Si^1,†^, Shuming Zhang^1,†^, Huiru Guo^1^, Wei Luo^1, 3^, Yuqin Feng^1^, Xinkang Du^1^, Fangzhi Mou^1*^, Huiru Ma^2,3*^, and Jianguo Guan^1,3*^*

1. State Key Laboratory of Advanced Technology for Materials Synthesis and Processing, International School of Materials Science and Engineering, Wuhan University of Technology, China

2. School of Chemistry, Chemical Engineering and Life Science, Wuhan University of Technology, China

3. Wuhan Institute of Photochemistry and Technology, China

^*^Address correspondence to: Fangzhi Mou; moufz@whut.edu.cn；Huiru Ma; mahr@whut.edu.cn and Jianguo Guan; guanjg@whut.edu.cn

†These authors contributed equally to this work

**The PDF file includes:**

Figs. S1 to S10

Tables S1 to S2

**Other Supplementary Material for this manuscript includes the following:**

Movies S1 to S5


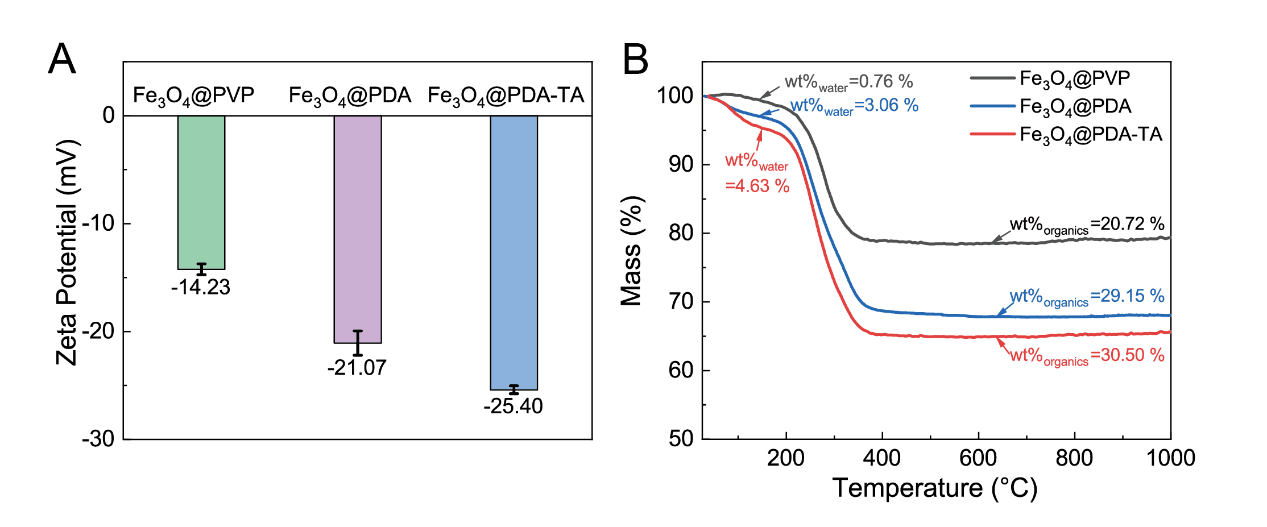


**Fig. S1. Zeta potentials and thermogravimetry (TG) analysis of Fe_3_O_4_@PVP, Fe_3_O_4_@PDA and Fe_3_O_4_@PDA-TA NPs.** (A) Zeta potentials and (B) TG curves of Fe_3_O_4_@PVP, Fe_3_O_4_@PDA and Fe_3_O_4_@PDA-TA NPs, respectively.

**
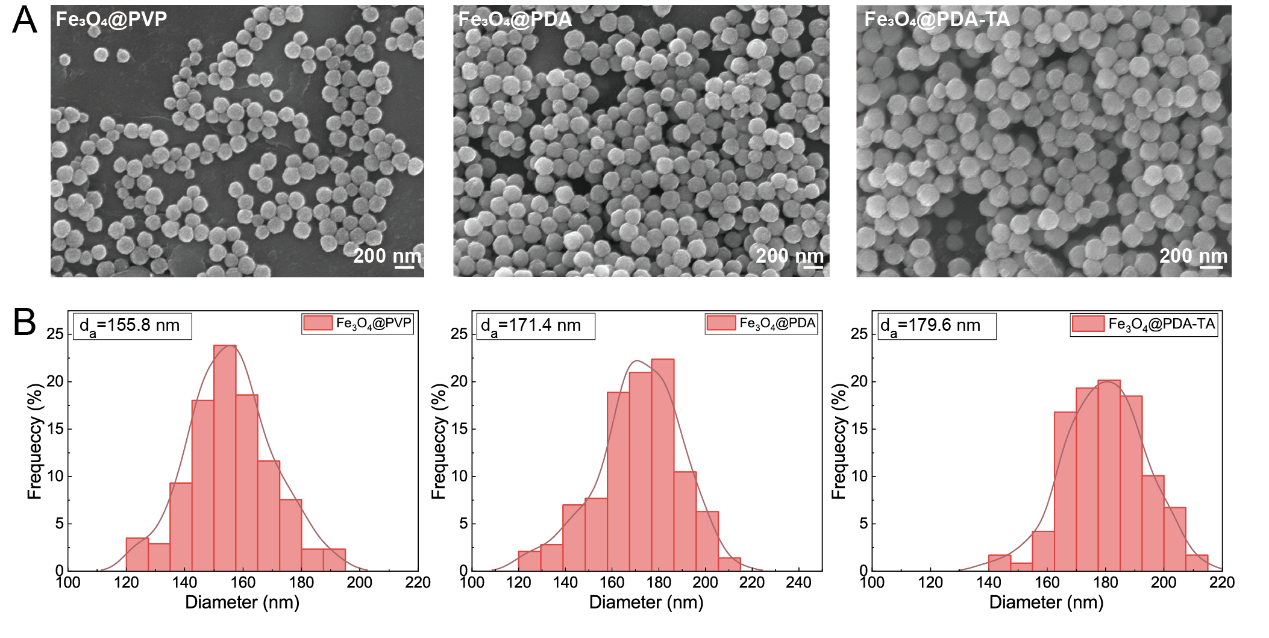
**

**Fig. S2. Average sizes of Fe_3_O_4_@PVP, Fe_3_O_4_@PDA and Fe_3_O_4_@PDA-TA NPs.** (A) SEM images in low magnification and (B) size distributions and average of particles diameters (d_a_) of Fe_3_O_4_@PVP, Fe_3_O_4_@PDA and Fe_3_O_4_@PDA-TA NPs.

**
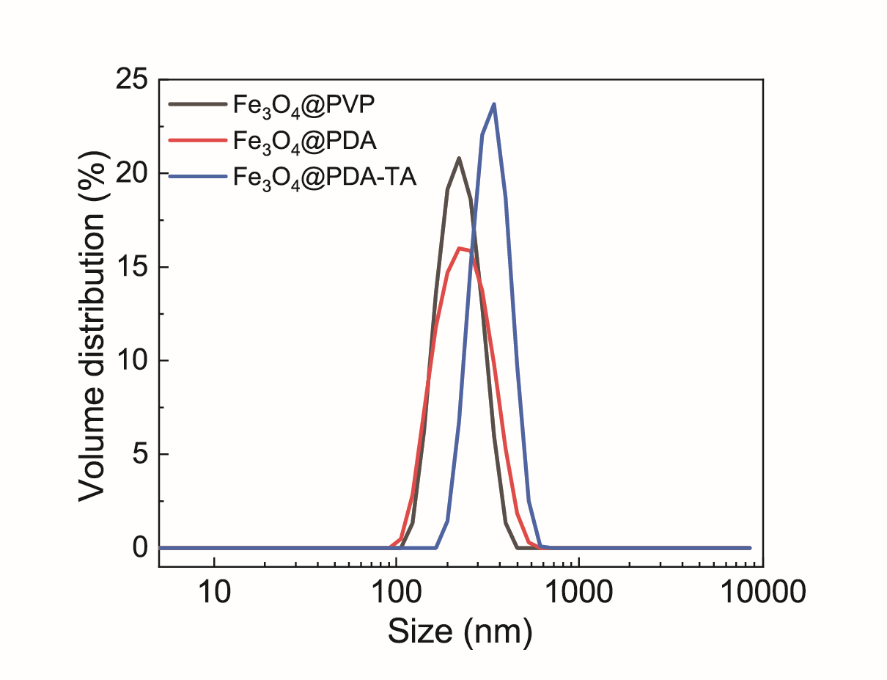
**

**Fig. S3. Hydrodynamic sizes of Fe_3_O_4_@PVP, Fe_3_O_4_@PDA and Fe_3_O_4_@PDA-TA NPs.** Hydrodynamic sizes of Fe_3_O_4_@PVP, Fe_3_O_4_@PDA and Fe_3_O_4_@PDA-TA NPs measured using the dynamic light scattering (DLS) method.


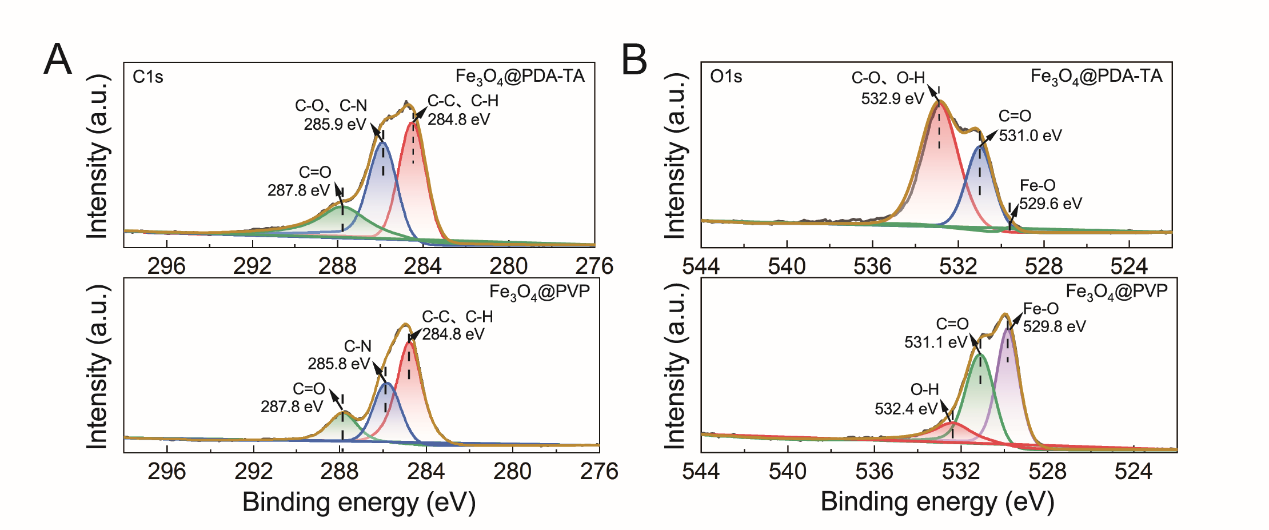


**Fig. S4. X-ray photoelectron spectroscopy (XPS) analysis of Fe_3_O_4_@PVP and Fe_3_O_4_@PDA-TA NPs.** High-resolution and deconvoluted XPS spectra of (A) C 1s and (B) O 1s of Fe_3_O_4_@PVP and Fe_3_O_4_@PDA-TA NPs.


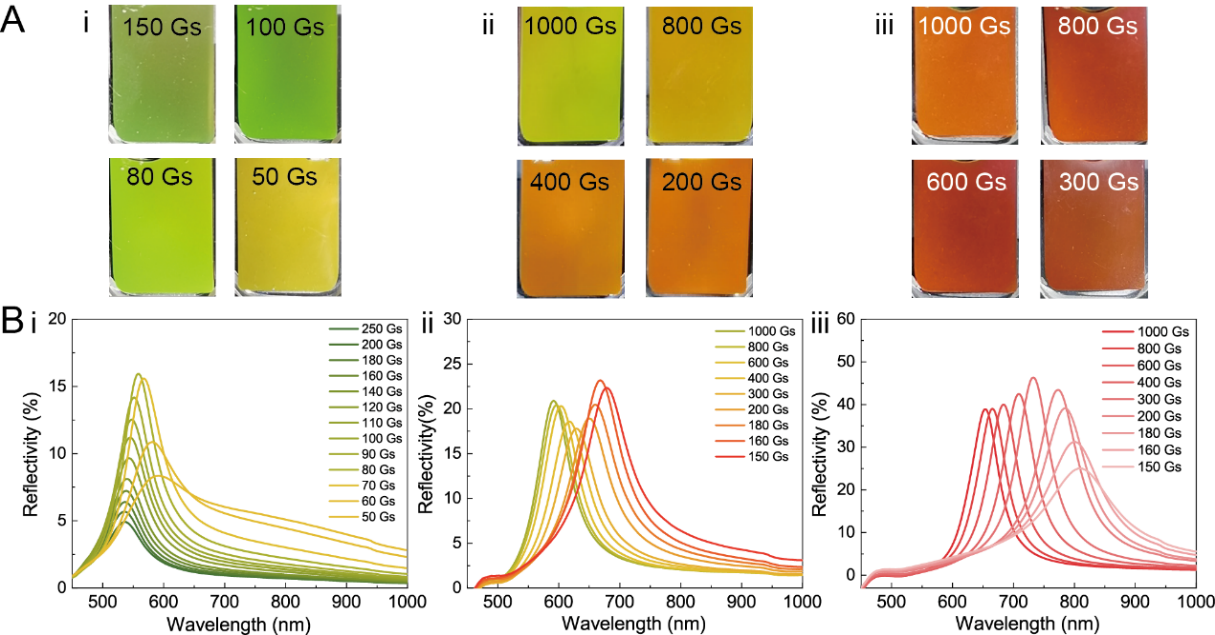


**Fig. S5. Magnetochromic performances of Fe_3_O_4_@PVP, Fe_3_O_4_@PDA and Fe_3_O_4_@PDA-TA NPs.** Digital photographs (A) and reflection spectra (B) of aqueous suspensions of Fe_3_O_4_@PVP (i), Fe_3_O_4_@PDA (ii) and Fe_3_O_4_@PDA-TA (iii) NPs under different magnetic-field strengths.


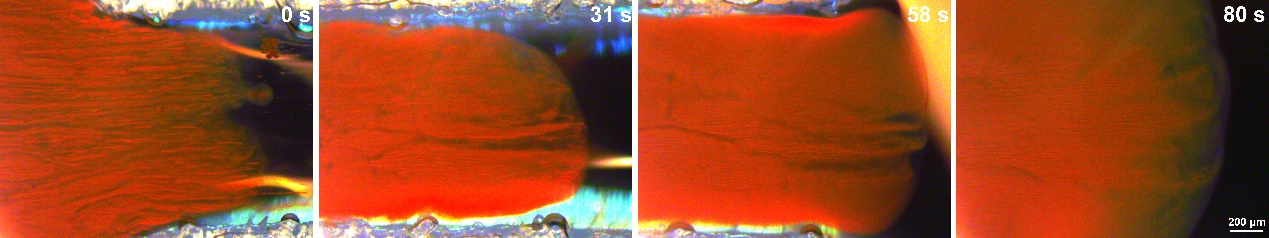


**Fig. S6.** **Structural colors of swarming Fe_3_O_4_@PDA-TA NRs.** Time-lapse dark-field microscopic images of swarming Fe_3_O_4_@PDA-TA NRs when driven by an **H**_r_(*t*).


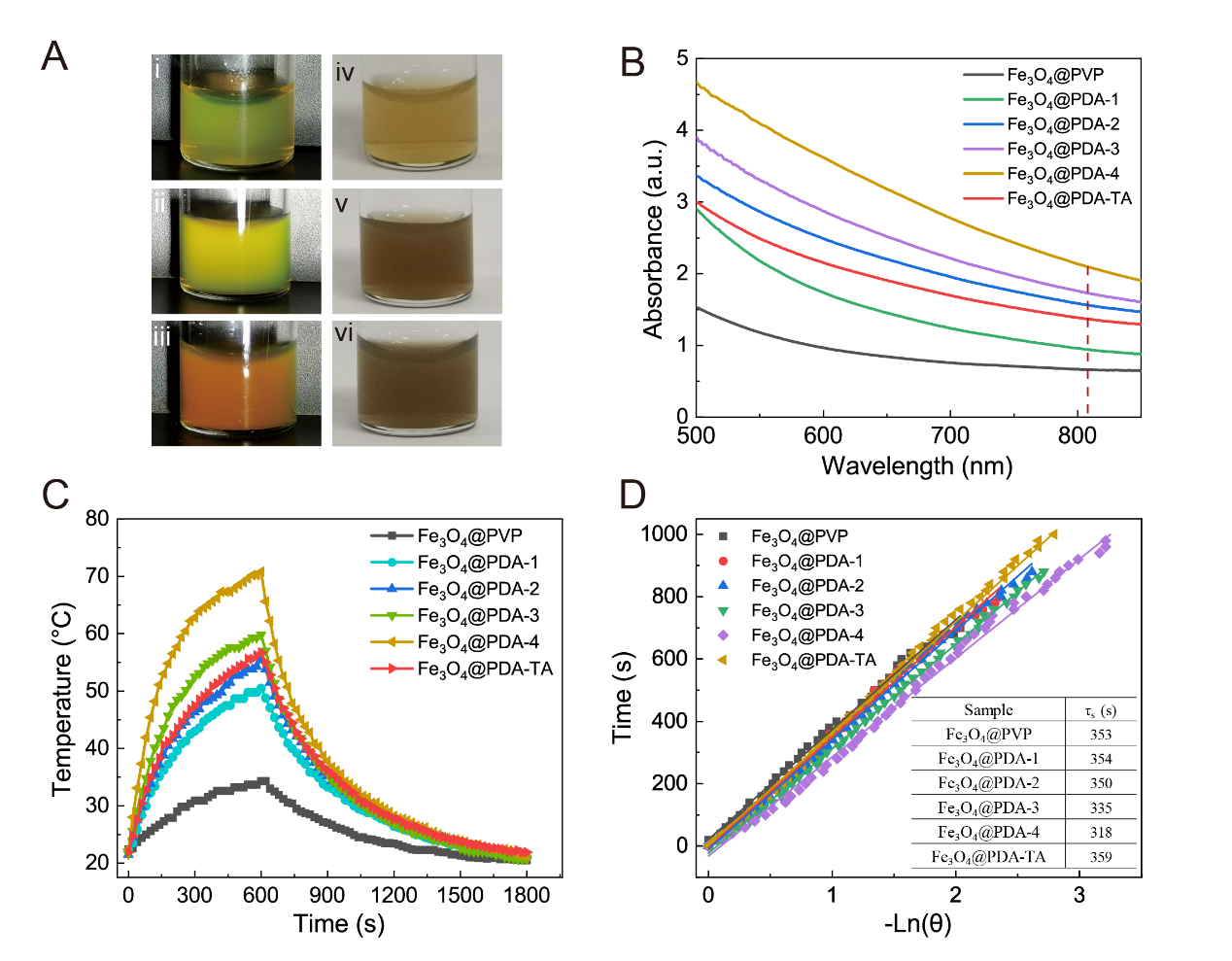


**Fig. S7.** **Optical properties, UV-Vis-NIR light absorbance and photothermal performances and of Fe_3_O_4_@PVP, Fe_3_O_4_@PDA and Fe_3_O_4_@PDA-TA NPs.** (A) Digital photographs of aqueous suspensions of Fe_3_O_4_@PVP (i, iv), Fe_3_O_4_@PDA (ii, v) and Fe_3_O_4_@PDA-TA (iii, vi) NPs with (the left column) or without (the right column) a 1000 Gs magnetic-field. (B) UV-Vis-NIR light absorption spectra, (C) photothermal heating curves, and experimental (colored dots) and (D) fitting plots (colored lines) of cooling time (*t*) versus negative natural logarithm of *θ* (-Ln (*θ*)) of aqueous suspensions of Fe_3_O_4_@PVP, Fe_3_O_4_@PDA-*i* (*i* = 1 to 4), and Fe_3_O_4_@PDA-TA NPs (*C*p = 125 μg mL^−1^). The power density (*I*) of the NIR laser (808 nm) was 1.0 W cm^−2^ in C, and the suspensions were heated for 600 s and cooled for 1200 s. The inset table in D shows the *τ*_s_ of each NPs, which equals the slope of the fitting lines.

The photothermal conversion efficiency (*η*) of Fe_3_O_4_@PVP, Fe_3_O_4_@PDA-*i* (*i* = 1 to 4) and Fe_3_O_4_@PDA-TA NPs was analyzed according to a widely reported method [*73*], in which the *η* was calculated by,

$\eta=\frac{hA\left( T_{\max}-T_{\mathrm{surr}} \right)-Q_{\mathrm{dis}}}{I(1-{10}^{-A_{808}})}$ (Equation S1)

where *h* is the heat transfer coefficient, *A* is the area of the sample being irradiated, 𝑇_max_ refers to the highest temperature in the heating period, 𝑇_surr_ is ambient temperature, 𝑄_dis_ refers to the heat absorbed by the NPs aqueous solutions and that dissipated to the surrounding, *I* is the laser power density (1.0 W cm^−2^) and 𝐴_808_ refers to the absorbance of the NPs aqueous solutions at 808 nm (Fig. S7A).

The "hA" in Equation S1 can be calculated by Equation S2,

$\tau_{s}=\frac{m_{D}c_{D}}{hA}$ (Equation S2)

where *m*_D_, *c*_D_ are the mass of deionized (D.I.) water (1.0 g) and heat capacity (4.2 J g^−1^), respectively, and *τ*_s_ is a constant. *τ*_s_ can be calculated by Equation S3,

*t* = -*τ*_s_ Ln (*θ*) (Equation S3)

where *t* is the time of the cooling period, the *θ* was defined as the driving force in the cooling process, as calculated by Equation S4,

$\theta=\frac{T-T_{\mathrm{surr}}}{T_{\max}{-T}_{\mathrm{surr}}} =$ $\frac{T-T_{\mathrm{surr}}}{\Delta T}$ (Equation S4)

in which *T* is the temperature in the cooling period and $\Delta T$ represents the maximum temperature variation of the suspensions in the heating period.

And *Q*_dis_ can be denoted as Equation S5,

$Q_{\mathrm{dis}}=\frac{m_{D}c_{D}\left( T_{max(water)}-T_{\mathrm{surr}} \right)}{\tau_{s(water)}}$ (Equation S5)

The temperature rising of D.I. water was 0.3°C (Fig. 4B) and thus the *Q*_dis_ could be neglected. Therefore, the *η* of the NPs was calculated by Equations S1-S5, and the results are shown in Table S2.


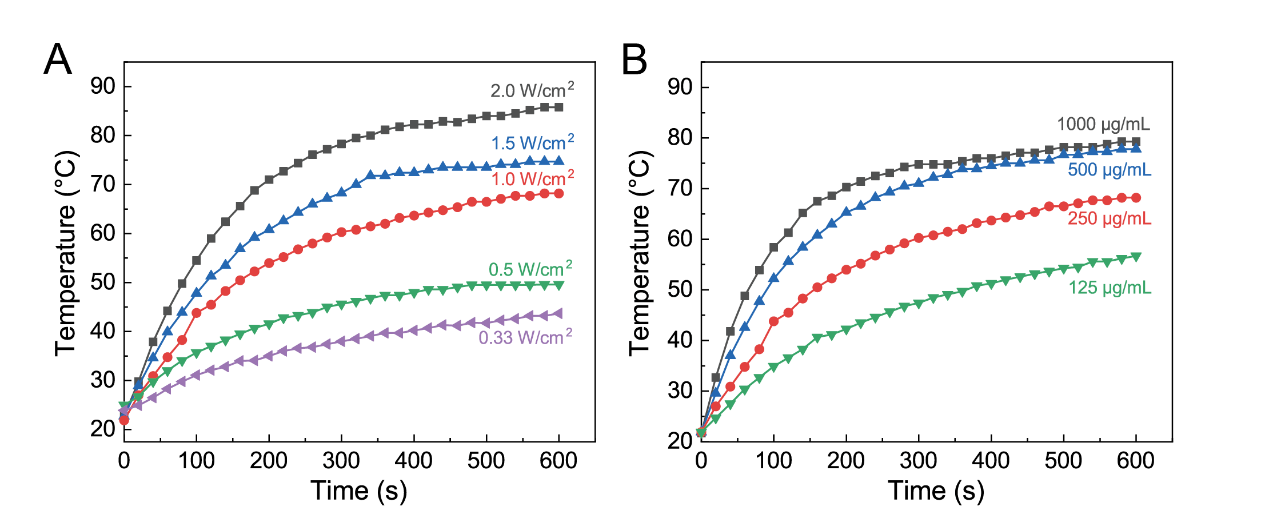


**Fig. S8. Photothermal heating curves of Fe_3_O_4_@PDA-TA NPs at different laser power densities (*I*) and different concentrations (*C*_p_).** (A) Photothermal heating curves of aqueous suspensions of Fe_3_O_4_@PDA-TA NPs at different *I* of 0.33, 0.5, 1.0, 1.5 and 2.0 W cm^−2^ and (B) at different *C*_p_ of 125, 250, 500 and 1000 μg mL^−1^. The *C*_p_ in A is 250 μg mL^−1^ and the *I* in B is 1.0 W cm^−2^.

**
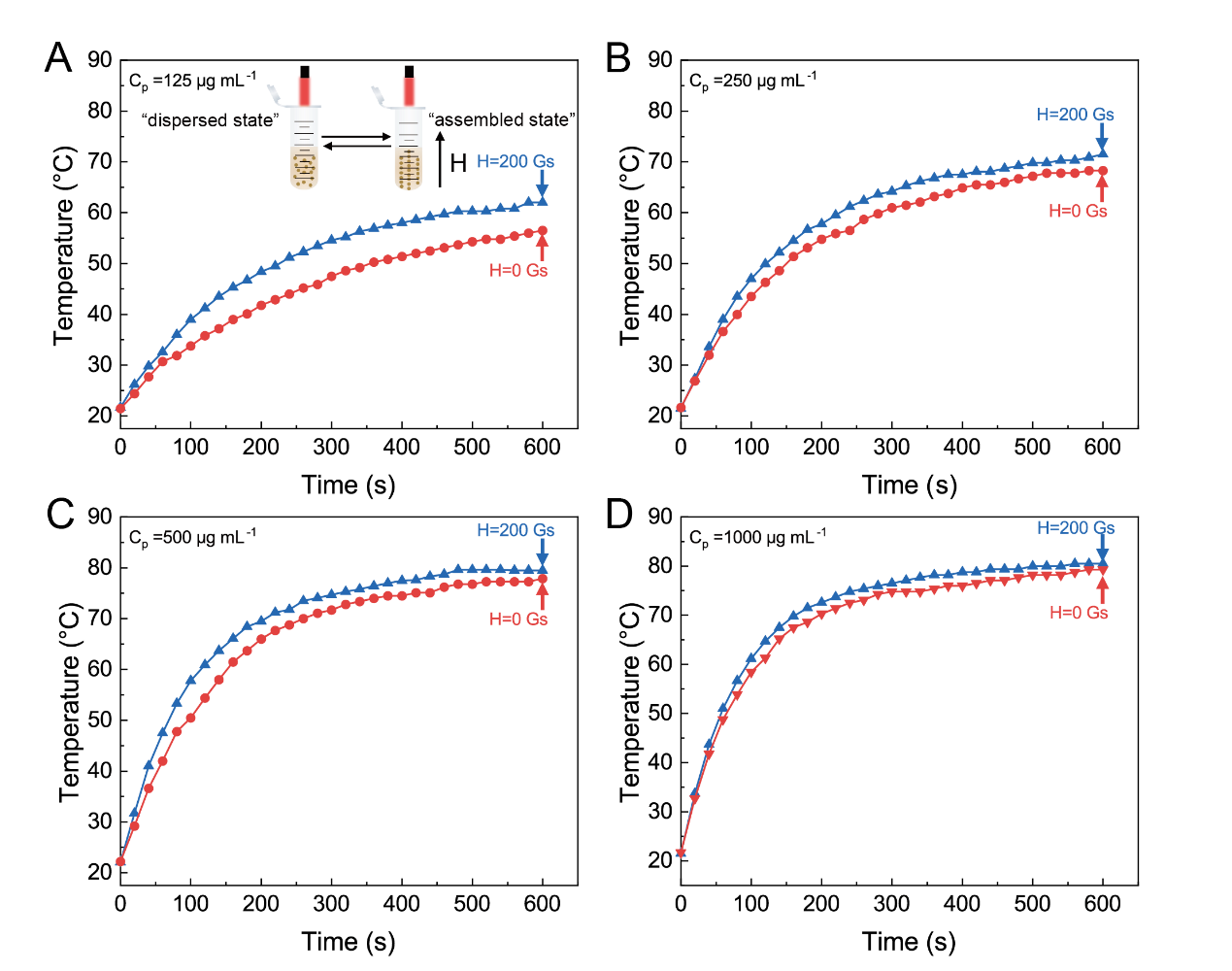
**

**Fig. S9. Photothermal heating curves of Fe_3_O_4_@PDA-TA NPs at different assembly states.** Photothermal heating curves of Fe_3_O_4_@PDA-TA NPs aqueous suspensions in the assembled state (blue curves) and dispersed state (red curves) at different *C*_p_ of (A) 125, (B) 250, (C) 500 and (D) 1000 μg mL^−1^, respectively. All suspensions were irradiated by a NIR laser with an *I* of 1.0 W cm^−2^.

**
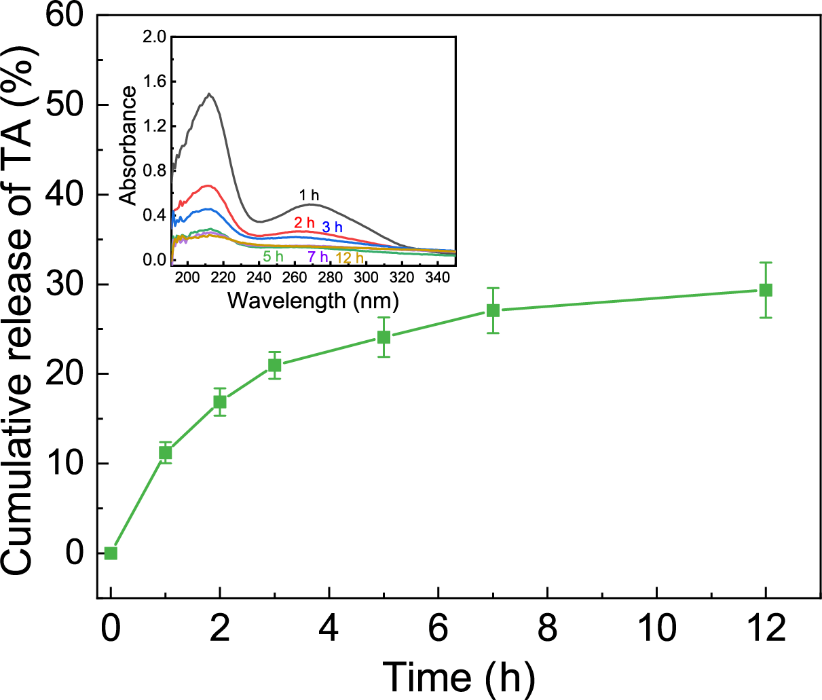
**

**Fig. S10. Cumulative release of TA from the Fe_3_O_4_@PDA-TA NPs over time.** The inset shows the corresponding UV-Vis-NIR absorbance spectra of supernatant of Fe_3_O_4_@PDA-TA NPs suspensions at different time points.

**Table S1. Mass concentrations of different elements in** **Fe_3_O_4_@PDA-TA and Fe_3_O_4_@PVP NPs from XPS.**

| Samples | Atomic content | | | | C/Fe | O/Fe |
| --- | --- | --- | --- | --- | --- | --- |
|  | Fe 2p (%) | C 1s (%) | O 1s (%) | N 1s (%) |  |  |
| Fe_3_O_4_@PDA-TA | 1.3 | 69.4 | 23.5 | 5.8 | 53.38 | 18.08 |
| Fe_3_O_4_@PVP | 11.2 | 52.9 | 31.3 | 4.5 | 4.72 | 2.79 |

**Table S2.** **The photothermal conversion efficiency (), *A*_808 nm_, and *T* of Fe_3_O_4_@PVP, Fe_3_O_4_@PDA-*i* and Fe_3_O_4_@PDA-TA NPs. Fe_3_O_4_@PDA-*i* (*i* = 1 to 4) represents the Fe_3_O_4_@PDA NPs prepared at different feeding concentration of DA (*C*_DA_) in the reaction solution.**

| Samples | *C*_DA_ (mg mL^−1^) | *A*_808 nm_ | Δ*T*_808 nm_ (°C) | *η* (%) |
| --- | --- | --- | --- | --- |
| Fe_3_O_4_@PVP | 0 | 0.663 | 11.9 | 18.09 |
| Fe_3_O_4_@PDA-1 | 0.17 | 0.943 | 28.3 | 37.90 |
| Fe_3_O_4_@PDA-2 | 0.33 | 1.560 | 34.0 | 41.96 |
| Fe_3_O_4_@PDA-3 | 0.67 | 1.730 | 38.2 | 48.80 |
| Fe_3_O_4_@PDA-4 | 1.33 | 2.096 | 48.4 | 64.44 |
| Fe_3_O_4_@PDA-TA | 0.33 | 1.366 | 34.8 | 42.54 |

**Supplementary Movies**

**Movie S1.** Reversible assembly and magnetic propulsions of single Fe_3_O_4_@PDA-TA NRs.

**Movie S2.** Swarming motions of Fe_3_O_4_@PDA-TA NRs when driven by an **H**_r_(*t*) and an **H**_p_(*t*).

**Movie S3.** Swarming motions of Fe_3_O_4_@PDA-TA NRs when driven by an **H**_r_(*t*) under dark-field microscopy.

**Movie S4.** Swarming Fe_3_O_4_@PDA-TA NRs actively targeting and covering a simulated superficial infection site utilizing their successive rolling and crawling collective motions and their subsequent photothermal bacterial treatment under NIR laser irradiation.

**Movie S5.** Swarming Fe_3_O_4_@PDA-TA NRs actively targeting a simulated deep-seated infection site by navigating through a zigzag narrow microtube.

References

73. J. F. Zhang, C. X. Yang, R. Zhang, R. Chen, Z. Y. Zhang, W. J. Zhang, S. H. Peng, X. Y. Chen, G. Liu, C. S. Hsu *et al.*, Biocompatible D-A Semiconducting Polymer Nanoparticle with Light-Harvesting Unit for Highly Effective Photoacoustic Imaging Guided Photothermal Therapy. *Adv Funct Mater.* 2017;27(13), 1605094.
